# Comparison of the Calibrated Automated Thrombogram Using Standard and Low Plasma Volumes in Dogs

**DOI:** 10.1111/vcp.70088

**Published:** 2026-03-07

**Authors:** Erin M. Phillips, Shauna L. Blois, Gabrielle Monteith, Anthony C. G. Abrams‐Ogg, R. Darren Wood, Benoît Cuq

**Affiliations:** ^1^ Department of Clinical Studies, Ontario Veterinary College University of Guelph Guelph Ontario Canada; ^2^ Department of Pathobiology, Ontario Veterinary College University of Guelph Guelph Ontario Canada; ^3^ Department of Small Animal Clinical Sciences, School of Veterinary Medicine University College Dublin Dublin Ireland

**Keywords:** canine, hematology, plasma volume, thrombin generation

## Abstract

**Background:**

Thrombin generation assessment using calibrated automated thrombography (CAT) requires a standard volume of plasma (80 μL) and reagent (20 μL) run in triplicate. A CAT method using lower plasma and reagent volumes could benefit veterinary patients as it lowers the sampling burden in patients.

**Objectives:**

To compare standard CAT methodology to a low plasma and low reagent volume CAT method in dogs.

**Methods:**

Platelet‐poor plasma samples were obtained by direct jugular venipuncture in dogs with low (*n* = 10), high (*n* = 10), and normal (*n* = 10) thrombin generation potential, recruited from a tertiary referral hospital. Method comparison was performed between standard CAT (80 μL plasma, 20 μL reagent‐ method 1) and low volume CAT (40 μL plasma, 10 μL reagent‐ method 2). Lag time (*lag time*); endogenous thrombin potential (*ETP*); peak (*peak*); and time to peak (*ttpeak*) were assessed on the thrombin generation curves.

**Results:**

There was excellent agreement between methods 1 and 2 for all parameters. Lin's concordance coefficients were 0.97, 0.94, 0.96, and 0.96 for *lag time, ETP, ttpeak*, *and peak*, respectively. There was a small bias for all parameters (*p* < 0.05), resulting in a significant change for *lag time* only. There was a significant predictive linear equation that, when modeled, allowed the conversion of method 2 to method 1 values. The methods had similar variation when measuring TG variables.

**Conclusions:**

Based on the results in this small group of dogs, we conclude that low‐volume CAT appears to be an alternative to the standard testing method in dogs.

## 1 Introduction

Thrombin is the key enzyme of blood coagulation, with procoagulant, prothrombotic, and anticoagulant functions, and the measurement of thrombin generation (TG) reflects a significant portion of the hemostatic function in the blood [1, 2]. The TG assay measures the potential of plasma to generate thrombin over time [3, 4]. The TG assay has been used to assess TG in healthy dogs and dogs receiving anticoagulant therapy [5, 6, 7, 8, 9, 10]. It has also been used to evaluate hemostasis in various disease states in dogs including hypercoagulability in hyperadrenocorticism, dogs with thrombosis, and in dogs with hemophilia A and B [11, 12, 13, 14, 15, 16, 17].

Calibrated automated thrombography (CAT) is a commercial, semi‐automated method of performing TG using whole blood, platelet‐poor plasma (PPP) or platelet‐rich plasma. The assay measures the ability of thrombin to cleave a fluorogenic substance. Typically, TG with CAT requires a volume of plasma (80 μL) and reagents: tissue factor (20 μL), thrombin calibrator (20 μL), and fluorescent substrate (20 μL), run in triplicate. A recent method comparison in people showed appropriate accuracy when measuring TG using half the volume of plasma (40 μL) and all reagents (10 μL) when compared to the standard protocol [18].

The low‐volume CAT method has potential use in veterinary patients, as the assessment of multiple hemostatic tests often requires large volumes of citrated plasma. The consequences of excess venipunctures and hospital‐acquired anemia are of particular concern in anemic or coagulopathic patients and those with small body weights [19, 20]. If low plasma volume CAT was found to provide comparable results to standard volumes in companion animals, it could decrease the sampling burden on an animal, thereby lowering the risk of developing hospital‐acquired anemia and potentially improving survival to discharge.

Moreover, TG is used only in veterinary medicine in a research setting. The need for large plasma volumes and specialized equipment, as well as the high cost of reagents, limits the use of CAT in clinical practice to date. Reducing the volume of reagents by half would lower the cost of future research involving the use of CAT. A technique using lower plasma volumes and cost of materials may also allow CAT to become more accessible outside of a research setting.

The objective of this study was to perform a method comparison between TG assays in dogs using the standard methodology vs. a half‐substrate‐half‐reagent (low volume) method, using CAT. We hypothesized that there would be no significant difference between CAT variables using standard sample and reagent volumes compared to the low volume method in dogs with a low, normal, and high TG potential.

## 2 Materials and Methods

### 2.1 Study Design

The study was performed using archived samples from dogs previously determined to have low (hypocoagulable), normal, and high (hypercoagulable) TG potential. Client consent was obtained before recruitment of patients into the study. The Institutional Animal Care Committee approved this research, and standards established by the National Council on Animal Care and the national Animals for Research Act (1980) were followed during this study.

Thrombin generation was assessed in all patients using both standard (80 μL) and low (40 μL) volumes of plasma in combination with standard (20 μL) and low (10 μL) volumes of each reagent. The standard volume and reagent protocol was termed method 1, and the low volume and reagent protocol was termed method 2.

### 2.2 Study Population

Healthy dogs (*n* = 10) with normal TG potential were recruited from staff and students of a tertiary referral hospital, and collected plasma banked for use in several different studies. Samples were collected from one dog in 2014 (stored at −80°C), and nine dogs in 2021–2022. Older samples were included based on previous research showing samples frozen at −80°C were stable for up to 12 months [7]. The dogs were between 1 and 7 years of age, and a range of breeds, sex, and body weights were included. Patients had no significant abnormalities found on physical examination, complete blood count, biochemistry profile, urinalysis and coagulation profile. There was no history of significant illness, and dogs received no medication other than parasite prophylaxis.

Samples from hypercoagulable dogs with high TG potential (*n* = 8) and hypocoagulable dogs with low TG potential (*n* = 10) were collected from the hospital caseload from 2020 to 2021. An additional 2 samples from hypercoagulable dogs were collected in 2014 (stored at −80°C). Dogs considered hyper‐ and hypocoagulable had a hematocrit > 0.30 L/L and a platelet count > 100 000 × 10^9^/L to prevent artefactual hyper‐ or hypocoagulability TEG readings, respectively [21, 22, 23].

The inclusion criterion for dogs with high TG potential was a diagnosis of hypercoagulability, based on one or more of the following: (1) diagnostic imaging findings consistent with thrombosis and/or (2) laboratory evidence of hypercoagulability based on thromboelastography MA and/or G value above the reference interval. Exclusion criteria included administration of anti‐coagulant agents within 24 h of testing or anti‐platelet agents within 7 days of testing.

Inclusion criteria for dogs with a low TG potential were one or more of the following: low MA and/or G on thromboelastography, prolongation of prothrombin time and/or activated partial thromboplastin time, and/or dogs currently receiving rivaroxaban anticoagulant therapy and considered to be within or above therapeutic levels (therapeutic rivaroxaban anti‐Xa activity was considered 150–250 ng/mL; Comparative Coagulation Laboratory, College of Veterinary Medicine, Cornell University) [24, 25].

### 2.3 Sample Collection and Storage

Healthy dogs underwent direct jugular venipuncture for collection of 2 mL EDTA blood, 2 mL whole blood for serum separation, and 10.4 mL into 3.2% sodium citrate evacuated tubes (1.8 mL tubes, 9:1 blood: citrate ratio‐ BD Vacutainer; Beckton Dickson, Franklin Lakes, New Jersey, USA). The collected blood was banked in aliquots to be used across several separate studies. The citrate tubes were inverted 10 times to ensure adequate mixing of citrate and blood. A free‐catch urine sample was also collected from healthy dogs. Samples from the healthy dogs were submitted for complete blood count, biochemistry profile, coagulation profile consisting of prothrombin time, activated partial thromboplastin time, and fibrinogen level (STA‐Compact, Diagnostica Stago, Parsippany, NJ) and urinalysis at a local reference laboratory (Animal Health Laboratory, University of Guelph).

Dogs with high and low TG potential underwent jugular venipuncture for routine diagnostic purposes. An additional 1.8 mL citrated blood was collected at that time for research purposes and available residual citrated plasma from other diagnostic tests was also banked when available.

Immediately after collection, citrated blood not submitted for the coagulation profile was centrifuged (3200 ***g*** for 15 min) to retrieve PPP. The PPP was divided into 0.5–1.0 mL aliquots and stored at −80°C for later TG analysis.

### 2.4 Calibrated Automated Thrombography (CAT) Assay

Thrombin generation measurement was performed using the CAT method on a Thrombinoscope (CAT Thrombinoscope, Diagnostic Stago, Asniere sur Seine, France) using software connected to an automated fluorometric microplate reader (Fluoroskan Ascent; Thermo Scientific, Waltham, MA, USA), as previously described [4]. Assays were performed using a 96‐well microplate (Immulon 2 HB; Thermo Scientific, Waltham, MA, USA).

Standard and low volume samples were analyzed on separate 96‐well plates, as the analyzer cannot dispense different volumes of fluorogenic reagent in the same plate. Samples from the same patient were allocated to the same plate, and the standard and low volume plates for that group of patients were performed sequentially on the same day. Testing order was assigned using a random team generator (https://pickerwheel.com/tools/random‐team‐generator). Before analysis, frozen citrated plasma samples were thawed in a 37°C water bath for 10 min, then vortexed at 1000*g* for 5 s. Plasma samples were analyzed within 30 min of thawing.

Distilled water was placed in the first row of each plate for each run as a negative control. A positive control was run once per plate using plasma from the same healthy normal dog (collected at the start of the trial and stored at −80°C until use). A PPP‐Reagent (PPP Reagent 5 pM; Thrombinoscope, Diagnostic Stago, Asniere sur Seine, France) and a thrombin calibrator (Thrombin calibrator TA 20.0; Thrombinoscope, Diagnostic Stago, Asniere sur Seine, France) were added to designated wells at 20 μL for standard volumes (method 1) and 10 μL for low volumes (method 2). A plasma volume of 80 μL was added to wells assessed with standard volume (method 1), and 40 μL of plasma was added to wells assessed with low volume (method 2). A buffer solution containing calcium chloride and a fluorogenic substrate (FluCa‐Kit; Thrombinoscope, Diagnostic Stago, Asniere sur Seine, France) at a volume of 20 μL (method 1) or 10 μL (method 2) was added to each well to initiate TG. Each sample was measured in triplicate.

The thrombin calibrator and fluorescence in each plasma sample were measured at 10 s intervals for a total of 60 min. A 10 s interval was chosen over the standard 20 s to account for faster coagulation time in dogs compared to humans [26, 27, 28]. Due to the shorter interval between measurements, only half the plate was used (48 wells) per run. Data parameters reported were lag time (*lag*), the time in minutes to generate 10 nM of thrombin; endogenous thrombin potential (*ETP*), the total enzymatic synthesis of thrombin measured as the area under the curve; maximal or peak generation of thrombin measured in nM (*peak*); and the time in minutes to reach the maximal concentration of thrombin (*ttpeak*).

### 2.5 Statistical Analysis

Lin's concordance, measurement and testing of bias, as well as regression questions were modeled to determine if method 2 agreed with method 1. Normality was tested using a Shapiro Wilk test on the paired differences. Data was log transformed to meet the assumptions of normality for all tests. In developing the predictive equations for method 2, both linear and quadratic effects were tested and quantified. The level of significance was set at *p* < 0.05. Method precision was evaluated by calculating assay variability (standard deviation divided by the mean of an individual's triplicate measurement, multiplied by 100). Coefficients of variation (CV) for each TG variable measured by method 1 and method 2 were checked for normality; only ETP met assumptions for normality. Wilcoxon signed‐rank tests and a paired *t*‐test were used to test for differences in CV of each TG variable between method 1 and method 2.

## 3 Results

**FIGURE 1:**
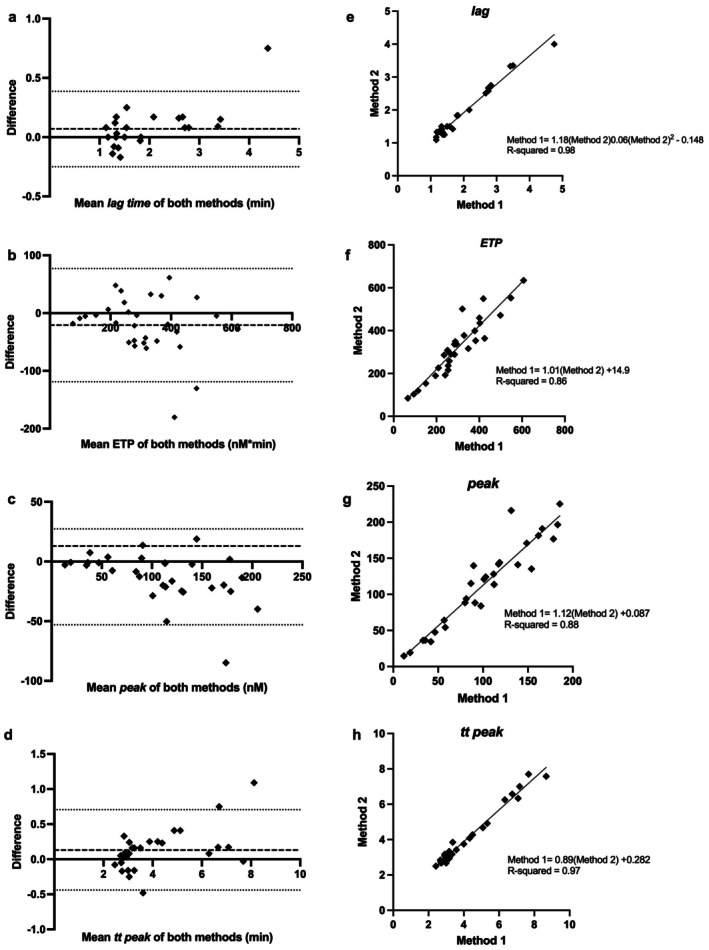
Bland–Altman plots and correlations of four thrombin generation parameters using a standard method using 80 μL plasma (method 1) and a low volume method using 40 μL plasma (method 2) for calibrated automated thrombogram measurement in 30 dogs (10 healthy, 10 hypercoagulable, 10 hypocoagulable). Bland–Altman plots were created using the difference between the two methods vs. the mean of the two methods, and results are shown for *lag* (a), *ETP* (b), *peak* (c), and *ttpeak* (d). Bias is indicated by the dashed line on each plot; upper and lower 95% confidence intervals are indicated with the dotted line. Lin's Correlation coefficient comparing methods 1 and 2 is shown for *lag* (e), *ETP* (f), *peak* (g), and *ttpeak* (h). *ETP* (endogenous thrombin potential), *lag* (lag time), *peak* (peak concentration of thrombin), and *ttpeak* (time to peak).

The TG results compared between method 1 and method 2 were similar for each parameter at the 25th, 50th, and 75th percentile (Table 1). There was excellent agreement between methods 1 and 2 for all TG parameters, as is demonstrated by the correlation graphs and Bland–Altman plots (Figure 1). Lin's concordance coefficients were 0.97, 0.94, 0.96, and 0.96 for *lag time, ETP, ttpeak*, and *peak*, respectively. There was a small bias for all parameters (*p* < 0.05), resulting in a significant change for *lag time* only. There was a significant predictive linear equation that, when modeled, allowed for the conversion of method 2 to method 1 values. Median coefficients of variation were calculated for the TG variables measured by methods 1 and 2. There were no significant differences between the coefficients of variation between methods 1 and 2 when measuring each TG variable (Table 2).

**TABLE 1:** Median and interquartile ranges of thrombin generation (TG) variables measured with the calibrated automated thrombogram (CAT) assay using the standard volume of 80 μL plasma (method 1) and a low volume method using 40 μL plasma (method 2) in 30 dogs (10 normal, 10 hypo‐, and 10 hypercoagulable).

TG parameter	Method	25% Percentile	Median	75% Percentile
*lag* (min)	1	1.3	1.5	2.2
	2	1.3	1.5	2
*ETP* (nM*min)	1	241	285	383
	2	227	312	399
*peak* (nM)	1	57.9	101.9	138.8
	2	64.3	118.1	144
*ttpeak* (min)	1	2.9	3.2	5.1
	2	2.8	3.2	4.7

*Note:* Method 1 = standard volume (80 μL plasma) CAT, method 2 = low volume (40 μL plasma) CAT; *peak*, peak thrombin generation; *ttpeak*, time to peak thrombin generation.

Abbreviations: *ETP*, endogenous thrombin potential; *lag*, lag time.

**TABLE 2:** Descriptive and comparative statistical analysis of thrombin generation (TG) variables measured with the calibrated automated thrombogram (CAT) assay using the standard volume of 80 μL plasma (method 1) and a low volume method using 40 μL plasma (method 2).

	Method 1		Method 2		*p*
	Median	Range	Median	Range	
CV *lag*	0	0–5.6	0	0–5.63	0.25
CV *ETP*	1.1	0.05–4.88	0.79	0.07–5.44	0.71
CV *peak*	2.31	0.1–7.42	1.16	0.04–13.47	0.39
CV *ttpeak*	1.96	0–3.7	1.04	0–8.56	0.58

*Note:* method 1 = standard volume (80 μL plasma) CAT, method 2 = low volume (40 μL plasma) CAT; *peak*, peak thrombin generation; *ttpeak*, time to peak thrombin generation.

Abbreviations: CV, coefficient of variation; *ETP*, endogenous thrombin potential; *lag*, lag time.

## 4 Discussion

This study described the use of low volume TG analysis using CAT in dogs with a range of TG potentials. The low volume CAT method was found to correlate well with the standard CAT method in normal, hypercoagulable, and hypocoagulable dogs. There was a bias noted in the lag time variable when the low volume method was used. However, as *ETP* is the main TG parameter typically referenced when assessing a dog's coagulation status, this bias in *lag time* is unlikely to be clinically relevant.

The results of the present study are similar to those found in people [18, 29]. In those studies, standard and low volume CAT methods were comparable in plasma samples from healthy people, people taking oral contraceptives, and people receiving vitamin K antagonists. In the present study, assay precision was not significantly different when CAT was performed using standard or low volumes. A previous study in people noted unacceptable assay precision and increased machine errors when using human plasma samples less than 40 μL [18]. While the present study did not evaluate assay performance in plasma samples lower than 40 μL, similar poor assay performance might be expected. However, being able to use a plasma volume of 40 μL vs. 80 μL would permit CAT to be performed in smaller samples, such as those collected from small patients or even aliquots from biobanks.

There were several limitations in this study. According to the Clinical & Laboratory Standards Institute Guidelines (https://clsi.org/media/1435/ep09a3_sample.pdf), method comparisons should be performed on 40 samples. This preliminary method comparison study did not reach that number, and therefore, future studies comparing standard and low volume CAT in a larger group of dogs would be beneficial. There is also a degree of variability in TG data between each run, with a reported TG inter‐assay variability of 12.9% in healthy beagles [5]. As the fluorescence substrate volume used in the CAT had to be the same for all samples within a run, normal and low volume samples could not be assessed within the same run. Though assay variability was controlled to some degree by running paired samples on the same day and on the same plate, the inability to perform CAT analysis on paired samples within the same run likely increased the variability in data between the two methods. Despite this limitation, an excellent correlation was found between both methods, which promotes a strong clinical utility of the low volume TG assay.

Another limitation of our study is that thrombin generation via CAT is no longer commercially available, which can make it difficult to directly apply the findings of the present research to currently available tests. However, newer methods of thrombin generation such as the ST Genesia use the same method principles, namely fluorogenic substrates in the activation of thrombin, as the CAT used in the present study. The ST Genesia has similar analytical variation in comparison to the CAT [30], which supports that concept that research data obtained using CAT methodology will likely be applicable to the ST Genesia. Our research findings may help guide future studies investigating low volume thrombin generation assays using the ST Genesia. Additionally, the overall concept of tailoring assays to suit the unique needs of different species is a valid theme in veterinary medicine that should continue to be explored. Our findings contribute to the current body of research investigating smaller sample volumes in veterinary patients and may encourage future studies of a similar nature.

This study demonstrated that the low volume CAT method produced comparable results to the standard protocol in dogs. This information could allow TG to be used more routinely in dogs with low blood volume, could lessen the sampling burden in anemic dogs, lower the risk of hospital‐acquired anemia and potentially improve survival to discharge. Thrombin generation via CAT is no longer commercially available. However, there are other thrombin generation analyzers commercially available that use a fluorogenic substrate and a similar platform to the CAT. Further research would be required to determine if the sample volume could be changed for other analyzers. A lower reagent volume would reduce the cost of future research using TG analysis.

## 5 Conclusions

Low volume CAT appears to be a valid alternative to the standard testing method in the present study of dogs with a range of TG potentials. Future studies comparing standard and low volume CAT in a larger population of dogs would be beneficial.

## Funding

This work was supported by OVC Pet Trust.

## Conflicts of Interest

The authors declare no conflicts of interest.
